# The Influence of Nd and Sm on the Structure and Properties of Sol-Gel-Derived TiO_2_ Powders

**DOI:** 10.3390/molecules26133824

**Published:** 2021-06-23

**Authors:** Albena Bachvarova-Nedelcheva, Stancho Yordanov, Reni Iordanova, Irina Stambolova, Angelina Stoyanova, Nelly Georgieva, Veronica Nemska

**Affiliations:** 1 Institute of General and Inorganic Chemistry, Bulgarian Academy of Sciences, Acad. G. Bonchev str., bld. 11, 1113 Sofia, Bulgaria; reni@svr.igic.bas.bg (R.I.); irinast@svr.igic.bas.bg (I.S.); 2 Institute of Metal Science, Equipment and Technologies “Acad. A. Balevski” with Center for Hydro- and Aerodynamics at the Bulgarian Academy of Sciences, 67 Shipchenski prohod str., 1574 Sofia, Bulgaria; stancho14@abv.bg; 3 Department Chemistry and Biochemistry, Faculty of Pharmacy, Medical University—Pleven, Kl. Ohridski str., 1, 5800 Pleven, Bulgaria; astoy@abv.bg; 4 Department Biotechnology, Faculty of Chemical and Systems Engineering, University of Chemical Technology and Metallurgy, Kl. Ohridski Blvd, 8, 1756 Sofia, Bulgaria; nelly.georgieva@yahoo.com (N.G.); vnemska@uctm.edu (V.N.)

**Keywords:** sol-gel, powders, thermal stability, X-ray diffraction

## Abstract

TiO_2_ nanopowders modified by Nd and Sm were prepared using the sol-gel technique. It was found by XRD analysis that the samples containing Sm are amorphous up to 300 °C, while those with Nd preserve a mixed organic-inorganic amorphous structure at higher temperatures (400 °C). The TiO_2_ (rutile) was not detected up to 700 °C in the presence of both modified oxides. TiO_2_ (anatase) crystals found at about 400 °C in the Sm-modified sample exhibited an average crystallite size of about 25–30 nm, while doping with Nd resulted in particles of a lower size—5–10 nm. It was established by DTA that organic decomposition is accompanied by significant weight loss occurring in the temperature range 240–350 °C. Photocatalytic tests showed that the samples heated at 500 °C possess photocatalytic activity under UV irradiation toward Malachite green organic dye. Selected compositions exhibited good antimicrobial activity against *E. coli* K12 and *B. subtilis*.

## 1. Introduction

Titanium dioxide (TiO_2_) remains one of the most promising materials for various applications such as self-cleaning, gas sensors, catalytic performance, and environmentally friendly photocatalyst [1,2,3]. Due to its high optical transparency, thermal stability, nontoxicity, chemical inertness, and environmentally friendly nature it is the preferred oxide [1,2]. However, its application in a visible light range of the solar spectrum is limited due to the wide band gap energy (3.2 eV) and in order to increase catalytic efficiency, stability and light absorption, it should be modified by doping of metals. Recent review papers reported additional progress towards the efficient application of this promising material in water and wastewater treatment under visible light [4,5]. In recent years, modification with some rare earth (RE) metals has proven to be an efficient method of improving the photocatalytic properties of TiO_2_ and broadening its absorption within the solar spectrum [3,6,7]. Moreover, it was found that the presence of RE ions slowed down the rate of the charge-carrier recombination processes [6].

According to published literature, RE-modified TiO_2_ can be prepared by a wide spectrum of methods, such as sol-gel [6,8], hydrothermal [9], solvothermal [10], electrospinning [11], co-precipitation [12], and electrochemical [13]. It is reported that from all the above methods, sol-gel has the greatest possibility of homogeneous distribution of dopant in the host material with a large surface area of TiO_2_ particles. Some authors found that combining hydrothermal treatments with the sol-gel method provided an alternative approach for preparing TiO_2_ thus preventing the agglomeration of the nanocrystals [14]. It was also established that the synthesis method significantly affects the structural, optical, luminescence and photocatalytic properties. At present, it has been established that TiO_2_ modified with Y^3+^, Pr^3+^, Er^3+^, and Eu^3+^ obtained through the hydrothermal method approach exhibited a higher photocatalytic activity, while the samples prepared via the sol-gel method approach yielded more luminescence when irradiated with 980 nm photons [15].

The rare earth elements such as neodymium (Nd), holmium (Ho), cerium (Ce), gadolinium (Gd) have been also used for doping TiO_2_ and these studies have been summarized by Daghrir [16]. The neodymium (Nd^3+^) ions are well known for improving photocatalytic activity by decreasing the energy band gap due to the transfer of charge between the TiO_2_ valence/conduction band and the 4f level in rare earth ions [15,16].

However, Sm-doped TiO_2_ has been also investigated [17,18,19,20,21]. Most of the studies concluded that Sm^3+^ ions increase the surface area but also enhance the photocatalytic activity under UV or solar light irradiation [18]. With reference to organic dyes, it was established that the presence of Sm is highly effective against Rhodamine B and Methyl orange with an optimal doping concentration of 0.5% [22,23,24].

It is well known that titanium dioxide (TiO_2_) has been extensively studied also with reference to antibacterial applications. Although several TiO_2_ nanocomposites with antimicrobial capabilities have been reported, they exhibit poor antibacterial activity in the visible light region due to the large band gap of TiO_2_ (3.2 eV) [25]. Some new approaches established that the rare earth doping of TiO_2_ resulted in antibacterial activities in the visible light region [26,27,28]. Relatively few papers [22,25] reported on the antibacterial performance of Nd-TiO_2_ and Sm-TiO_2_ nanopowders but additionally modified by coating with Ag.

In our previous papers, we presented experimental studies concerning sol-gel synthesis of binary and multicomponent composite powders with good photocatalytic and antibacterial properties [29,30,31,32,33]. Our studies on modification with some lanthanides (La^3+^, Ce^3+^), TiO_2_ showed that doping with rare metals sometimes improved the photocatalytic activity under UV irradiation and was not beneficial under Vis irradiation [34,35,36]. On the basis of the accumulated knowledge, we continue extensive studies on the modified TiO_2_ powders investigating their thermal, optical, and structural properties. Our priority is to research new combinations from compositions containing TiO_2_, especially those that have not been investigated until now.

To our knowledge, a comparison of the sol-gel-derived TiO_2_ powders modified with Nd^5+^ and Sm^3+^ has not been reported until now. Hence, the focus of the present work is to pay more attention to the synthesis as well as the structural, optical, photocatalytic activities, and antibacterial properties of the obtained powdered samples. The properties of as-prepared samples were compared with pure TiO_2_ as well.

## 2. Results and Discussion

### 2.1. Phase Transformations 

**Figure 1:**
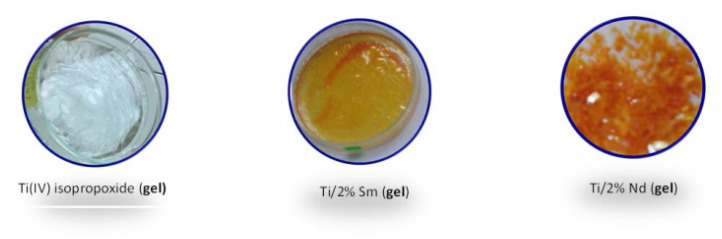
Images of the as-prepared gels.

All gels prepared at room temperature were transparent as the modified with Sm and Nd exhibited identical bright orange color and their visual observations are shown in Figure 1.

Bearing in mind the strong complexation processes occurred after the addition of acetylacetonate during the sol-gel processes it could be suggested that the observed color is due to the simultaneous presence of chelating agent and the rare eart ions Sm^3+^ and Nd^3+^.

**Figure 2:**
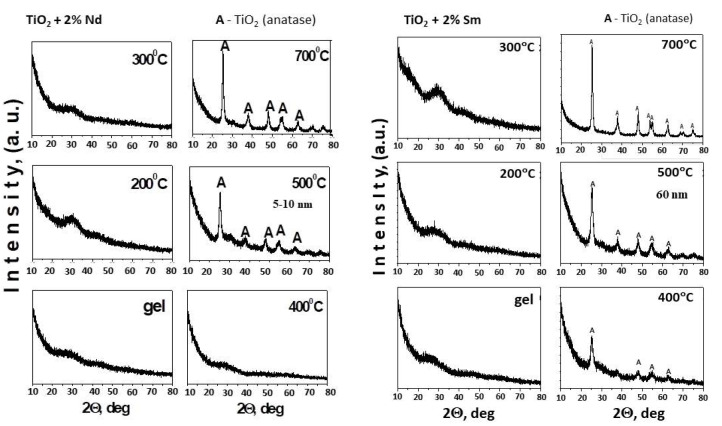
XRD patterns of TiO_2_ modified with 2% Nd and 2% Sm heat-treated at different temperatures: (A) TiO_2_-anatase.

**Figure 3:**
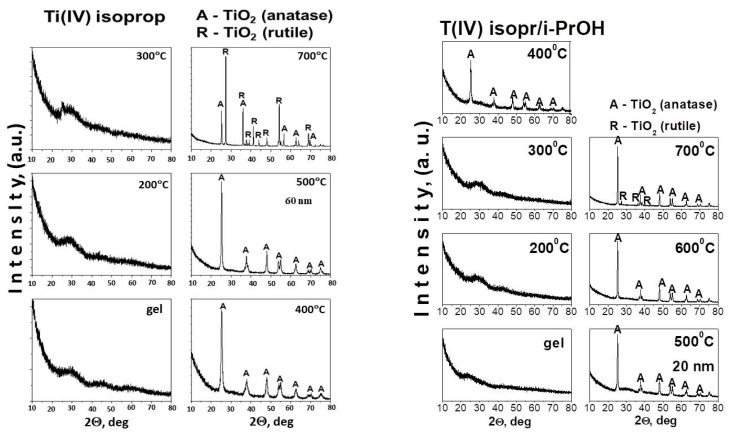
XRD patterns of used precursor Ti(IV) isopropoxide (TTIP) and TTIP dissolved in i-PrOH: (A) TiO_2_-anatase, (R) TiO_2_-rutile.

The XRD patterns of the gels and the heat-treated samples in the temperature range of 200 °C–700 °C are shown in Figure 2. The XRD patterns of the precursors used (TTIP and TTIP/i-PrOH) were presented for comparison, as well (Figure 3). As can be seen from Figure 2, both samples preserve the amorphous state up to 300 °C but the TN sample containing 2% Nd is amorphous even at 400 °C. Obviously, doping with Nd suppresses the earlier crystallization of TiO_2_ (anatase). Increasing the calcination temperature leads to the appearance of the first diffraction peaks at 2Θ = 25.3, 38.4 and 48.1 corresponding to TiO_2_ (anatase) (JCPDS 78-2486). There is an absence of peak at 2Θ = 27.4, which corresponds to TiO_2_ (rutile), which indicates that anatase is the only crystalline phase detected in the temperature range of 500–700 °C in both samples. It is worth noting that the phase transition TiO_2_ (anatase)-TiO_2_ (rutile) did not occur in the investigated samples (Figure 2) and both rare earth ions hinder this transformation. No characteristic peaks for Nd and Sm are observed in the modified samples, indicating that these ions do not substitute Ti^4+^ in the crystal lattices, which can be attributed to the difference in the radii of Ti^4+^ (0.53 Å), Nd^3+^ (0.99 Å) and Sm^+3^ (0.96 Å) [37,38]. Similar results have been obtained by Bokare et al. [25] which have suggested that possibly Nd and Sm exist in the form of Nd_2_O_3_ and Sm_2_O_3_ small particles, uniformly dispersed between the TiO_2_ nanocrystallite or deposited on the surface of TiO_2_ nanoparticles. From Figure 2, it can be also seen that at 500 °C, the main diffraction peak of anatase phase is broad and this indicated that the samples have a small particle size.

Looking at the XRD pattern of the pure TTIP and TTIP/i-PrOH (Figure 3), it could be seen that both undoped samples up to 300 °C exhibited similar behavior to TN and TS samples, but the difference is that the first TiO_2_ (anatase) crystals appeared at 400 °C while at 700 °C, TiO_2_ (rutile) was registered.

At 500 °C, the average crystallite size (calculated using the Sherrer’s equation, based on the strongest peak) of TiO_2_ (anatase) for sample TN is about 5–10 nm, while for the TS one it is about 60 nm (Figure 2). In comparison, at this temperature, the average crystallite size of TiO_2_ (anatase) is about 20 nm (Figure 3) in the undoped TTIP and TTIP + i-PrOH samples. Obviously, the Nd dopant preserves the smaller TiO_2_ (anatase) particles size dimensions. This refers to that the doping ions (Nd^3+^) retard the grain growth of TiO_2_ nanoparticles and similar results of decreasing crystalline nature of material were observed by several authors [22,25].

Our results concerning the calcination temperature effect on the phase formation concur with those reported in the literature [3,39,40,41]. The specific surface areas (SBET) of samples TN and TS were measured, and they are 118 and 81 m^2^/g, respectively. In comparison, the specific surface area of pure TiO_2_ obtained by the Ti(IV) isopropoxide is 21 m^2^/g. This higher value of the specific surface area for both samples could predict potential good environmental applications.

### 2.2. Thermal Stability of the Gels

**Figure 4:**
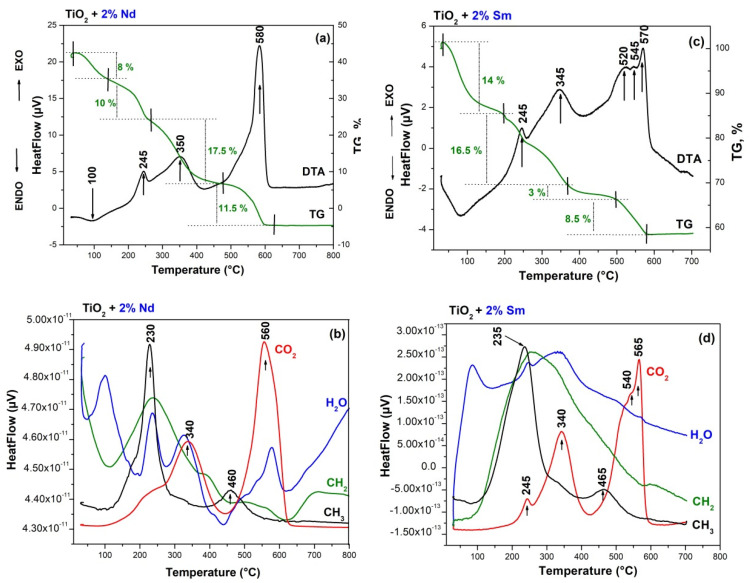
Differential thermal analysis (DTA)/thermogravimetric (TG) curves of the TN) (**a**,**b**) and TS (**c**,**d**) samples.

The thermal stability of gels aged at room temperature was investigated by simultaneous thermogravimetric (TG) and differential thermal analysis (DTA). DTA/TG curves of the used precursors are not presented but it has been studied and discussed elsewhere [42,43,44]. The DTA/TG curves are presented for both gels—TN (TTIP-2%Nd) and TS (TTIP-2% Sm) (Figure 4) and several stages could be marked on them. The DTA curves of both samples showed similar behaviour. As is seen from figure one of the common features is a stepwise release of the organics probably as a result of the higher number of organic groups due to the presence of solvent and chelating agent as well. The first decomposition step of the gels is a weak endothermic effect near 100 °C (Figure 4a–d). This step could be attributed to the evaporation of physically adsorbed water and/or organic solvent (isopropanol). The average mass loss after dehydration is about 10% for both samples. The first exothermic peak is at about 245 °C and it could be related to the combustion of alkoxide groups bonded to Ti-atom. That peak is accompanied by the mass loss of ~10% for the TN gel, while for the TS sample this value is higher (~16.5%). The next exothermic effect in both samples is at about 350 °C and it could be assigned to the combustion of residual organic groups but in the TS sample, it may be connected to the beginning of the TiO_2_ (anatase) crystallization as well. The mass loss at this stage is about 17% for the TN sample and ~10% for the TS one. As is seen there is a difference in the thermal behavior of the investigated samples above 500 °C. The comparison of the DTA-TG curves of the samples showed that one exothermic effect at about 580 °C is observed in the TN gel while in the other one, three consecutive exothermic effects were detected (at 520 °C, 545 °C, and 570 °C). It is also obvious that in both cases, a mass loss of an average of 10% is observed. Obviously, in the presence of samarium, the last decomposition is more gradual in comparison to the other sample. On the basis of these experimental facts, it could be assumed that the effects in the range 520–540 °C in the TS sample could be related to the oxidation of residual carbon and release of CO_2_. The last exothermic effects at 570 °C (for TS) and 580 °C (for TN) could be associated with the intensive crystallization of anatase for both cases (Figure 4). The results obtained by DTA correspond well to the XRD data (Figure 2), as well as to the results obtained by other authors for Nd and Sm-modified TiO_2_ powders [43,45,46].

### 2.3. Structural Studies and Optical Properties

**Figure 5:**
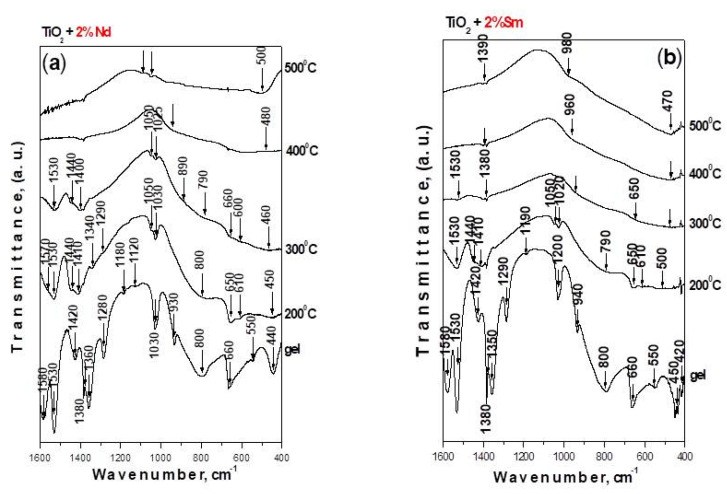
IR spectra of Ti(IV) isopropoxide modified with (**a**) 2% Nd and (**b**) 2% Sm.

The IR spectroscopy was used not only for verifying the phase transformations occurring with heat treatment (in the temperature range 200–500 °C) but also to evaluate the rate and degree of hydrolysis and condensation processes. The IR spectra of investigated samples are depicted in Figure 5. The vibrational spectra of pure Ti(IV) isopropoxide (TTIP) as well as of isopropanol were shown and discussed already elsewhere [42,47]. By analogy with our previous papers, the assignments of the vibrational bands of separate structural units are made on the basis of well-known spectral data for TTIP, isopropanol and crystalline TiO_2_ (anatase) [42,47]. Looking at Figure 5, it is seen that intensive bands are observed in the IR spectra of the gels, but their intensities decreased with the temperature increasing. Moreover, it is obvious that there is a similarity regarding the position and intensity of the bands for the heat-treated samples in the range 200–500 °C. A decrease in the intensity of organic groups is registered after heating in the range 200–300 °C. These groups disappeared completely above 300 °C. Generally, the bands located between 1500–1300 cm^−1^ are assigned to the bending vibrations of CH_3_ and CH_2_ groups. The band at 1120 cm^−1^ is characteristic for the stretching vibrations of Ti-O-C, while those at 1190 and 1020 cm^−1^ are assigned to the vibrations of terminal and bridging C-O bonds in alkoxy ligands [47]. The absorption bands below 1000 cm^−1^ in the samples correspond to C-H, C-O and deformation Ti-O-C vibrations [48,49]. In our previous investigations [50,51], it was found that the absorption region 1100–1020 cm^−1^ is very complex due to the overlapping of the vibrations of different structural units from the alkoxide and solvent. Inspite of that, many authors [52,53,54] use these bands for the interpretation of the degree of hydrolysis-condensation processes. Bearing in mind the similarity of the spectra of investigated samples, it is difficult to evaluate the completeness of the hydrolysis–condensation reactions. However, the absence of a weak band at about 1120 cm^−1^ in the IR spectra of TS gel gives us reason to suggest that more completed hydrolysis reactions occurred in that sample. The bands below 800 cm^−1^ correspond to the vibrations of TiO_6_ units [52,53]. Bearing in mind that the typical Sm-O and Nd-O stretching vibrations are in the range of 510–430 cm^−1^ [55,56] overlapping between the inorganic structural polyhedra is suggested.

#### UV-Vis Spectroscopy

**Figure 6:**
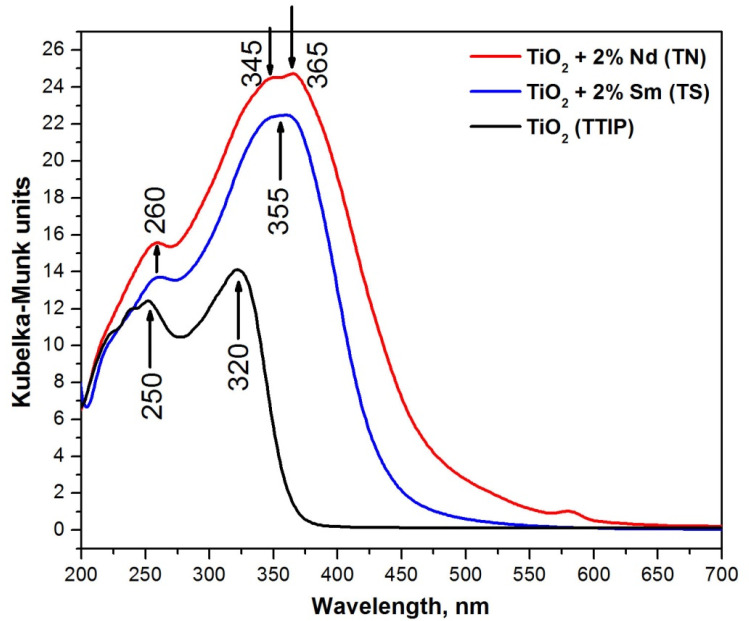
DR UV-Vis spectra of TN, TS, and pure Ti(IV) isopropoxide gels.

Aiming to evaluate the completeness of the hydrolysis–condensation processes, as well as to gain additional structural information, UV-Vis spectroscopy has been applied. Figure 6 shows the spectra of as-prepared TN and TS gels, which are compared to those of TiO_2_ gel obtained from Ti(IV) isopropoxide. The interpretation of the UV-Vis spectra is made on the basis of literature data as well as our previous results obtained in various systems containing TiO_2_ [21,44,49,52,53,54,57]. In Figure 6, several peaks could be distinguished: for undoped TiO_2_—250, 320 nm while for the Sm- and Nd-modified TiO_2_—260 nm as well as one broad band in the region 345–365 nm.

As it is known, the isolated TiO_4_ units exhibited the ligand-to-metal charge transfer band in the region 200–260 nm, while in a titania network (anatase), the charge transfer in TiO_6_ groups is above 300 nm data [49]. During the hydrolysis–condensation processes, coordination geometry is changed and as a result, polymerized Ti species (Ti–O–Ti links between TiO_6_ units) are formed [49,52,53,54,57]. Hence, an increase in the UV absorption peak above 300 nm (instead of that at 250–260 nm) occurred. Bearing in mind the higher intensity of the bands above 300 nm in comparison to these below 300 nm for the Sm and Nd-modified TiO_2_ samples, it could be suggested that the hydrolysis-condensation processes are more complete in these samples in comparison to the undoped TiO_2_. Moreover, the widening of the band in the range 345–365 nm could be also attributed to the f-f transitions of Sm^3+^ or Nd^3+^ [58,59].

UV-Vis spectroscopy was also used in order to investigate the optical properties of the investigated samples (Table 1). As can be seen, a red shift of the absorption edge in the Sm- and Nd-doped TiO_2_ powders (431.98 nm—2% Sm and 474.24 nm—2% Nd) is clearly observed in comparison to pure TiO_2_ gel (367.69 nm). It is obvious that the TN sample exhibited a higher cut-off value (474.24 nm) and increased absorption in the Vis region. The red shift in the doped samples can be ascribed to the charge transfer between the TiO_2_ valence band and 4f levels of modified ions (Nd^3+^ and Sm^3+^) [21].

Another peculiarity of the UV-Vis spectrum of TN gel is the presence of a weak peak in the visible region (~580 nm) which could be attributed to the f-f electronic transition to Nd^3+^ in the coordination environment of TiO_2_ nanostructures [60,61]. The calculated optical band gap values (*E_g_*) of pure and modified TiO_2_ gels are 3.37, 2.90, 2.61 eV, respectively (Table 1). Our findings correlate well to those obtained by other teams [22,25,62].

### 2.4. Photocatalytic and Antibacterial Properties

#### 2.4.1. Photocatalytic Activity

**Figure 7:**
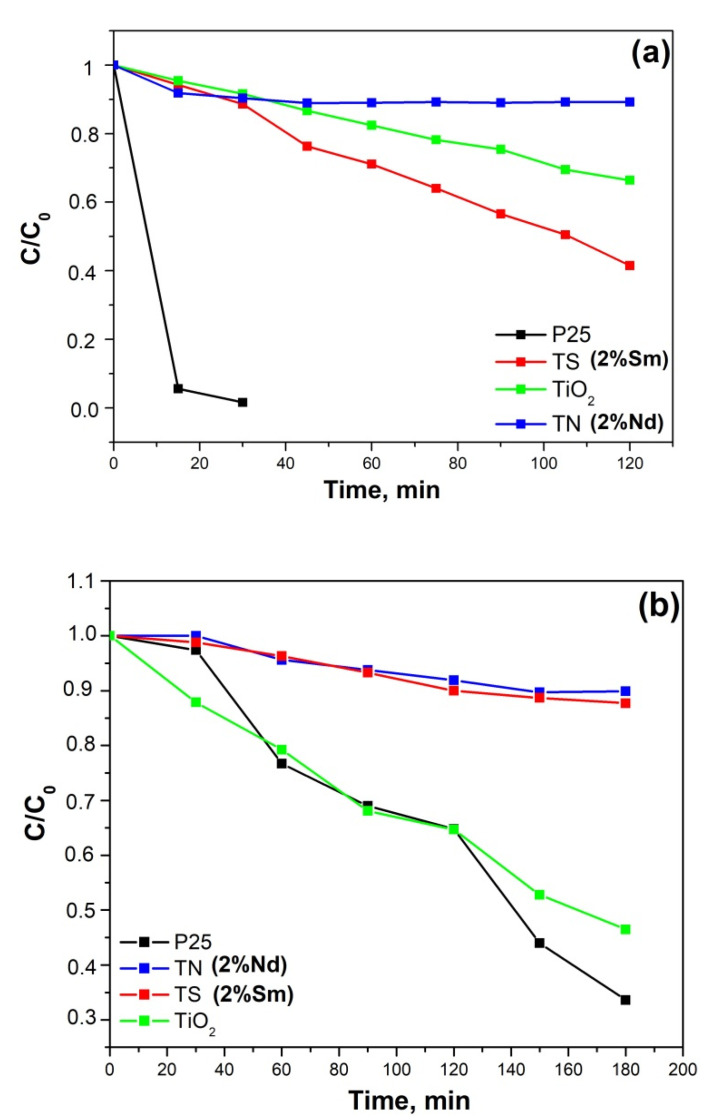
Photocatalytic activity against Malachite green of both samples TiO_2_/2%Nd and TiO_2_/2%Sm compared to pure TiO_2_ obtained by metal alkoxide and Degussa P25: (**a**) under UV irradiation; (**b**) under Vis irradiation.

The photocatalytic action of pure and modified TiO_2_ powders heated at 500 °C for 1 h was tested for degradation of MG dye water solution illuminated with UV and visible light. As it is well known, MG was selected as a model pollutant because of its intensive use in industrial processes. The degradation ratios (C/Co) of MG as a function of time for all samples were investigated and it is represented in Figure 7a,b. Blank tests of the photodegradation of the dye in the absence of a photocatalyst indicate that the photolysis can be ignored as less than 2% of MG was removed after 2 h illumination under UV or visible light. The MG dye degradation was also followed in the presence of Degussa P25 TiO_2_ in order to evaluate the photocatalytic ability of synthesized powders in comparison with the key photocatalyst (Figure 7a,b). It was found that the photocatalytic performance under UV light of sample TS is better than that of TN and pure TiO_2_ samples under the same conditions (Figure 7a). It is believed that small particle size and large specific surface area could be beneficial for photocatalytic activity. The particle size of the Nd-doped TiO_2_ was found to be about 10 nm (Figure 2). However, as the particle size is lowered below a certain limit, surface recombination processes can become dominant because of the increased surface-to-volume ratio and an optimum particle size for maximum photocatalytic efficiency exists [63]. Probably the lower photoactivity of the sample TN could be explained by the small size of its particles.

Bearing in mind the obtained experimental results it could be suggested that the specific surface area has little effect on the photocatalytic activity. Despite its lower specific surface area (81 m^2^/g) sample TS exhibited better photocatalytic properties in comparison to TN one (118 m^2^/g). According to Sun et al. [8], the possible reason could be the crystallization of TiO_2_ (anatase) at the earlier temperature of 400 °C while the TN sample preserved the amorphous state at the same temperature, as shown in Figure 2. As was already found, the anatase phase can contain more adsorbed water and hydroxyl groups on the surface of titania which helps to improve the photocatalytic activity of TS sample [8]. These data compare well with those obtained by XRD where it was shown that the Nd doping hinders earlier growth of TiO_2_ (anatase) particles (Figure 2).

The results of the photocatalytic test under illumination with visible light (Figure 7b) reveal that dopping with Nd and Sm was not beneficial for the degradation of the MG dye at our experimental conditions. As can be seen in Figure 7b, the photocatalytic performance of synthesized TiO_2_ under visible light is comparable to that of the best commercial photocatalyst Degussa P25.

**Figure 8:**
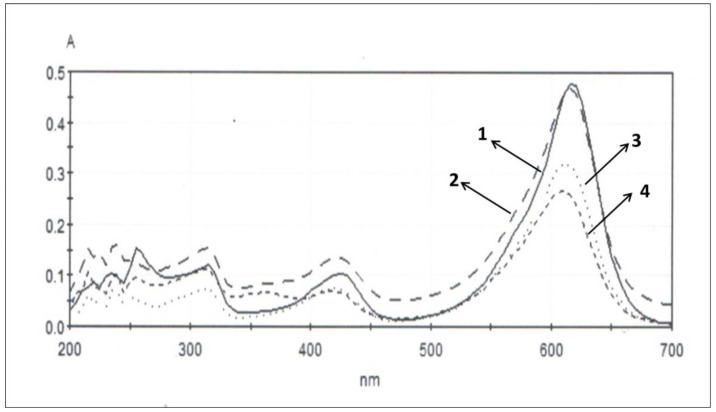
Change in the absorbance spectra of MG with the illumination time in the presence of synthesized by metal alkoxide TiO_2_ after: 0 min (**1**); 60 min (**2**); 120 min (**3**); 180 min (**4**) of Vis irradiation.

The absorbance spectra of MG versus visible light illumination time for the synthesized photocatalyst are shown in Figure 8. As irradiation time increases the height of peak at 618 nm decreases, which is a result of photocatalytic degradation of MG.

It is well-documented in the literature that the initial step in the TiO_2_-mediated photocatalysed degradation involves the generation of electron-hole (e−/h+) pair, leading to the formation of hydroxyl radical (•OH) and superoxide radical anion (O_2_•^−^). It has been suggested that these radicals are the primary oxidizing species in the photocatalytic oxidation processes and are highly reactive to attack the organic molecules [1,2,3]. The literature confirms that the appropriate dopping influences the photoactivity by electron or hole traps. When such a trap can cause formation of highly reactive species, the dopant introduction has a positive effect. If dopant introduction cannot decrease electron-hole recombination, such modification is ineffective in the degradation process [64,65].

The disagreements under visible and UV irradiations can be explained by whether the dopants act as electron and hole trappers or as recombination center of both the charges.

It can be suggested that the lower photocatalytic efficiency of doped samples in this study can be explained by the prevalence of the recombination processes.

As it is known, the photocatalytic activity of doped TiO_2_ depends on many factors, such as synthesis procedure, amount of dopant, light source, particle size, surface area, etc. Obviously, at our experimental conditions, the modification with ions of Nd and Sm did not impove the photoactivity under visible light illumination.

#### 2.4.2. Antibacterial Activity

**Figure 9:**
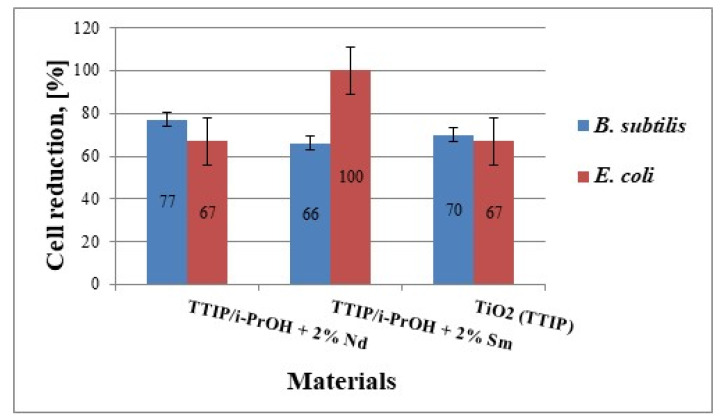
Cell reduction of *B. subtilis* NBIMCC 3562 and *E. coli* NBIMCC K12 by TiO_2_/2% Nd, TiO_2_/2% Sm and pure TiO_2_. Data are expressed as means ± SD (n = 3). Error bars denote the standard deviations of three trials.

The antibacterial activity of the investigated materials was tested against strains *B. subtilis* NBIMCC 3562 and *E. coli* NBIMCC K12 by measuring the inhibition zones formed around the materials and monitoring the dynamic of bacterial growth in their presence in a liquid medium. As well, the antibacterial properties of samples TS and TN were compared to those of the sol-gel-derived TiO_2_. The growth-inhibiting effect of the materials against the two bacterial strains was shown in Figure 9. Compared to the control samples (without materials added), all samples exhibited good antibacterial activity against both bacterial strains but their behavior during the analyses is different. It is worth noting that the TN sample showed the highest growth inhibition (about 77%) against the Gram-positive *B. subtilis* NBIMCC 3562, while the TS one demonstrated 100% growth inhibition against the Gram-negative *E. coli* NBIMCC K12 (Figure 8). The other two samples showed similar antibacterial activity (about 70% growth inhibition) towards both bacterial strains.

**Figure 10:**
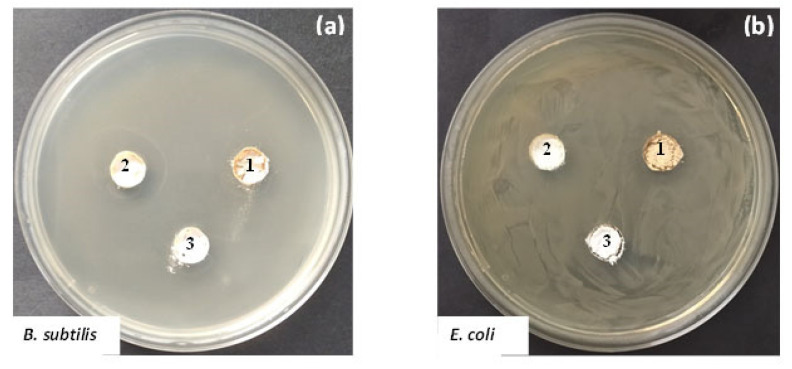
Antibacterial properties of samples TiO_2_/2% Nd (1), TiO_2_/2% Sm (2) and.pure TiO_2_ (3) obtained by metal alkoxide against *B. subtilis* NBIMCC 3562 (**a**) and *E. coli* NBIMCC K12 (**b**).

Another approach applied to estimate the antibacterial properties of the investigated samples refers to the measurement of the zones, which are free of bacterial growth. The obtained results revealed well-formed inhibition zones around materials, containing TS, TN and undoped TiO_2_. Comparison between results obtained showed that materials exhibited higher antibacterial activity against *B. subtilis* NBIMCC 3562 than *E. coli* NBIMCC K12. Also, the TS (2% Sm) sample demonstrated the highest antibacterial activity against *B. subtilis* NBIMCC 3562 (inhibition zone = 20 mm), whereas the lowest one was observed in the pure TiO_2_ sample (inhibition zone = 13 mm) (Figure 10). The highest inhibition zone against *E. coli* NBIMCC K12 was observed in the TN (2% Nd) sample (inhibition zone = 14.5 mm). The pure TiO_2_ sample again showed the lowest antibacterial activity against the Gram-negative test microorganism (inhibition zone = 11 mm).

The high antibacterial activity of the modified with Sm and Nd TiO_2_ samples could be explained with their lower bandgap which enhances the visible light absorption ability. On the other hand, their relatively higher surface area also results in the formation of highly reactive oxygen species (ROS) which are responsible for bacterial cell damage. Our results compare well to those obtained by other authors [25].

## 3. Materials and Methods

### 3.1. Materials and Reagents

Ti(IV) isopropoxide—TTIP (>98%, Merck, Darmstadt, Germany) Neodymium oxide, Nd_2_O_3_ (99,9 %, Janssen Chimica, Antwerpen, Belgium), Samarium oxide, Sm_2_O_3_ (Janssen Chimica, Anwerpen, Belgium 99.9%) and isopropanol, i-PrOH (>99.5 %, Merck, Darmstadt, Germany) have been used as main precursors for the obtaining titania powders. The acetylacetonate (AcAc, Sigma-Aldrich, Darmstadt, Germany) was used as a chelating agent to form stable complexes with TTIP. Commercial Degussa P25 TiO_2_ powder was kindly donated by Evonik Industries AG. All the reagents used were of analytical grade and were used without further purification.

### 3.2. Preparation of Nd and Sm-Modified TiO_2_ Gels

The experimental conditions for obtaining the initial solutions consist of several steps. The first solution was prepared by mixing of TTIP, i-PrOH and AcAc with vigorous stirring while keeping the molar ratio TTIP/C_3_H_7_OH/AcAc = 1:30:1 [66]. Several drops of nitric acid were added to obtain a clear solution. During the procedure, the molar ratio of AcAc/TTIP >2 was preserved. The obtained sol was transparent, with an orange color indicating the retention of the AcAc ligand in the xerogel [67]. The other solution was obtained by Nd_2_O_3_ or Sm_2_O_3_ dissolved in 1.5 mL HNO_3_ and isopropanol. Finally, both solutions were mixed with vigorous stirring. During the experimental procedure, no additional water was added. The sol-gel hydrolysis reaction was accomplished only in the presence of air moisture. The pH of the resulting solution was measured and found equal to 4–5. The gels’ ageing was performed in air for several days in order to allow further hydrolysis. Aiming to verify the phase transformations, all gels were subjected to stepwise heating in air from 200 °C to 700 °C for 1 h exposure time at each temperature value. The investigated samples were denoted as follows: (TTIP, TTIP/i-PrOH, TN (TTIP-2%Nd) and TS (TTIP-2%Sm). It has to be mentioned that the dopant concentration has been chosen based on several reasons: i.e., the literature survey on the TiO_2_ doping, our previous investigations [42,43] as well as the necessity of satisfactory performance against degradation of the selected organic dye and antibacterial test.

### 3.3. Characterization

The thermal stability of selected gels was determined by differential thermal analysis (LABSYSTM EVO apparatus) with Pt-Pt/Rh thermocouple at a heating rate of 10 K/min in air flow, using Al_2_O_3_ as a reference material. The accuracy of the temperature was ±5 °C. Heating of the samples was limited up to 600 °C. Gases evolved (EGA) during the thermal treatments were analyzed by mass spectrometry (MS) with a Pfeiffer OmniStarTM mass spectrometer (Pfeiffer Vacuum Technology AG, Wetzlar, Germany). Mass spectra recorded for TTIP and TTIP/i-PrOH (Figure 4b,d) show *m*/*z* of 14, 15, 18 and 44 ascribed to CH_2_, CH_3_, H_2_O and CO_2_, respectively. Powder XRD patterns were registered at room temperature with a Bruker D8 Advance diffractometer using Cu-K_α_ radiation. The specific surface area of samples heat-treated at 500 °C was measured using BET analysis (Quantachrome Instruments NOVA 1200e apparatus, Anton Paar GmbH, Graz, Austria). The optical absorption spectra of the powdered samples in the wavelength range 200–1000 nm were recorded by a UV-VIS diffused reflectance Spectrophotometer “Evolution 300” (Thermo Electron Corporation, Madison, WI, USA) using a magnesium oxide reflectance standard as the baseline. The absorption edge and the optical band gap were determined following Dharma et al. instructions [29]. The band gap energies (*E_g_*) of the samples were calculated by the Planck’s equation, where *E_g_* is the band gap energy (eV), *h* is the Planck’s constant, *c* is the light velocity (m/s), and *λ* is the wavelength (nm). The infrared spectra were registered in the range 1600–400 cm^−1^ using the KBr pellet technique on a Nicolet-320 FTIR spectrometer with 64 scans and a resolution of ±1 cm^−1^.

(1) $$E_{g}=\frac{h\cdot c}{\lambda }=\frac{1240}{\lambda }$$

### 3.4. Photocatalytic Experiments

The photocatalytic activities of pure and modified with Nd and Sm TiO_2_ powders were characterized by photodegradation of the dye malachite green (MG) as a model pollutant. For the degradation experiments, the initial concentration of the MG aqueous solution was 5 ppm. A fixed amount of 100 mg of each catalyst was added to 150 mL dye solution to form suspension and the suspensions were sonicated for 10 min. Before irradiation, the suspensions were magnetically stirred in the dark for 30 min in order to establish an adsorption-desorption equilibrium of the dye on the catalyst surface. The time at which the light was turned on was noted as starting point (*t* = 0) of the reaction at which time the concentration of the dye was denoted as *C_o_*.

Irradiation with UV-light was provided by a black light blue lamp (Sylvania BLB 50 Hz 8W T5, (Erlangen, Germany) with the major fraction of irradiation occurring at 365 nm. The lamp was fixed at 10 cm above the solution surface. The visible light source was a 500 W halogen lamp (Sylvania, Erlangen, Germany) fixed at 40 cm above the treated solution. All photocatalytic tests were performed at a constant stirring rate (450 rpm) and room temperature of 25 °C. Sampling was performed at regular intervals during the reaction. In order to separate the supernatant liquid from the solid particles the collected aliquot samples of the dye mixtures were centrifuged at 5000 rpm for 10 min. The phototocatalytic degradation of the dye was monitored by measuring the absorbances of clear supernatant aliquots by Jenway 6505 UV-Vis (designed and manufactured in UK) and Jenesys 10S UV-Vis spectrophotometers (made in China, design by USA) at the maximum absorption wavelength of MG—618 nm. The experimental data are the average from two or three measurements differing from each other by about 5%.

### 3.5. Antibacterial Assay

#### 3.5.1. Test Microorganisms, Media and Culture Conditions

The bacterial strains Bacillus subtilis NBIMCC 3562 and Escherichia coli NBIMCC K12 were selected as test microorganisms and were obtained from the National Bank for Industrial Microorganisms and Cell Cultures (NBIMCC, Bulgaria). Exponential cultures (OD 610 nm = 1.9) of both strains were obtained in Nutrient broth (NB)/Luria-Bertani (LB) broth, after cultivation in a shaker-incubator ES-20/60 (Biosan, Riga, Latvia, 120 rpm) at 30/37 °C for 24 h.

#### 3.5.2. Antimicrobial Activity Assay

The bacterial growth-inhibiting effect of the tested materials was investigated by studying the reduction of viable cells after exposure to them. To this end, 100 μL strain suspension of *B. subtilis* NBIMCC 3562 or *E. coli* NBIMCC K12, pre-adjusted to the turbidity of a 0.5 McFarland standard, and 10 mg of each material were added to flasks, containing 100 mL NB or LB broth, respectively. Samples, containing only bacterial cells (without materials added), were used as controls. The incubation process was performed on a shaker-incubator (120 rpm) at 30/37 °C for 24 h. The antibacterial activity of the investigated materials was determined by cell counts, calculated from the colonies, grown on NB/LB agar after 24 h of incubation at 30/37 °C and expressed as a percentage of cell reduction, according to Bachvarova-Nedelcheva et al. [33]. All tests were performed in triplicates and the results obtained showed the mean values.

In addition, the antibacterial activity of the materials against the two test microorganisms was also determined by the agar-well diffusion method [68]. Sterile NB/LB agar plates were inoculated with an exponential culture of the test strains, according to the spread-plate method. The investigated materials in the amount of 100 mg were loaded onto the marked wells in the agar plates and were then cultured in an incubator (Binder, Germany) at 30/37 °C for 24 h. The antibacterial activity was assessed by measuring the diameter of the obtained inhibition zones. Three replicates were made from each sample and the results show the mean values.

## 4. Conclusions

Transparent samarium- and neodymium-modified titania gels are prepared from Ti(IV) isopropoxide. The key role of the chelating agent (AcAc) for obtaining gels on the ground of Ti(IV) isopropoxide and isopropanol is confirmed. The presence of neodymium stabilizes the amorphous state of the sample up to higher temperature (400 °C) as compared to the other one containing samarium. It was established that the neodymium and samarium doping hinders the anatase-to-rutile phase transition and enhances the stability of the anatase phase at higher temperature values (700 °C). The DTA revealed that in the presence of samarium, the organic combustion occurs at higher temperatures (of 340 °C) when compared to that of neodymium (of 250 °C). The UV-Vis results showed that the investigated gels exhibited a red shifting of the cut-off in comparison to the pure sol-gel-derived TiO_2_ gel. Using IR spectroscopy, it was found that a more completed hydrolysis reaction occurred in the TiO_2_ sample modified with samarium. The photocatalytic activity under UV light of modified with Sm sample was better than that of Nd-modified and synthesized pure TiO_2_ sample. The doping with Nd and Sm was not beneficial for the degradation of the MG dye under visible light. However, the photoactivity of synthesized TiO_2_ under the same conditions was comparable to that of the commercial photocatalyst Degussa P25. The materials obtained demonstrated good antibacterial activity against *B. subtilis* 3562 and *E. coli* K12. It is found that the Sm-modified TiO_2_ is more sensitive against *B. subtilis*, while Nd-doped TiO_2_ exhibited 100% reduction of cells against *E. coli*.

## Figures and Tables

**Table 1:** Observed cut-off and calculated optical band gap values (*E_g_*) of the obtained gels.

Gels Composition, mol %	UV-Vis Results	
	*E_g_*, eV	Cut-Off, nm
TiO_2_ (Ti(IV) isopropoxide)	3.37	367.69
TS (2% Sm)	2.90	431.98
TN (2% Nd)	2.61	474.24

## Data Availability

The data presented in this study are available on request from the corresponding author.

## Sample Availability

Not available.
